# Carcinosarcoma of the Ovary Arising in the Background of a Mucinous Neoplasm Distinct From a Mural Nodule

**DOI:** 10.7759/cureus.110464

**Published:** 2026-06-08

**Authors:** Rachael Bare, Mohamed Alshal, John Diks, Logan Lawrence, Waqas Mahmud

**Affiliations:** 1 Pathology, Marshall University Joan C. Edwards School of Medicine, Huntington, USA

**Keywords:** gyn pathology, mucinous ovarian neoplasm, mural nodules, ovarian carcinosarcoma, ovarian tumors

## Abstract

Carcinosarcoma of the ovary (CSO) is a rare, aggressive biphasic neoplasm characterized by the simultaneous presence of malignant epithelial and mesenchymal components. Mural nodules, on the other hand, are circumscribed nodules in primary ovarian mucinous neoplasms that may harbor sarcoma-like, sarcomatous, or anaplastic elements. We describe a case of a 59-year-old female patient with CSO, originating in the background of cystic mucinous neoplasm, showing features distinct from a mural nodule. The case highlights diagnostic considerations and overlapping features of these complex ovarian tumors.

## Introduction

Carcinosarcoma of the ovary (CSO) is a rare malignant biphasic neoplasm that typically presents at an advanced stage in the sixth or seventh decade of life [1]. Grossly, they are large in size and have a morphology similar to their uterine counterparts. The malignant epithelial component is usually a high-grade carcinoma, whereas stromal components show sarcomatous features with or without heterologous elements [2]. 

Rarely, ovarian mucinous neoplasms may also display biphasic histology with the presence of a mural nodule that may harbor sarcomatous or anaplastic elements [3]. Sarcomatous nodules show various types of sarcomas, including undifferentiated sarcoma, while nodules of anaplastic carcinoma have variable appearances, including rhabdoid, epithelioid, and sarcomatous features [4]. We describe a case of CSO originating in the background of a mucinous neoplasm of the ovary, distinct from a mural nodule.

## Case presentation

**Figure 1:**
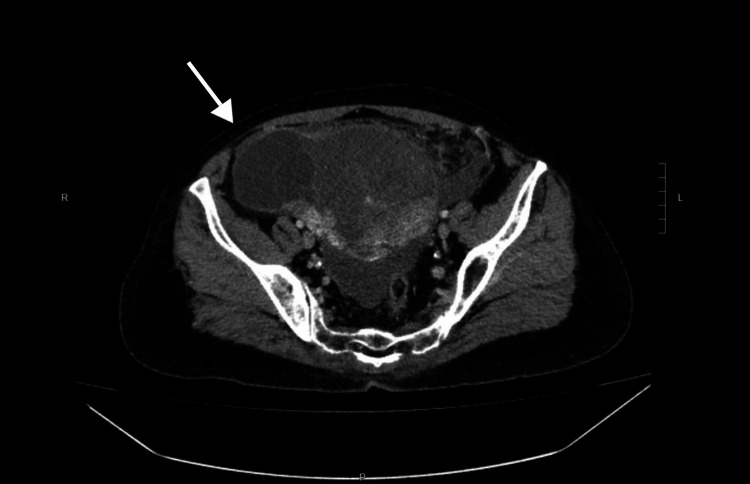
The CT scan showing a 15-cm pelvic mass.

The patient was a 59-year-old postmenopausal female with symptoms of right lower abdominal pain and distention for three months. She had no family history of gynecologic cancer, and her cancer antigen 125 (CA-125) levels were within normal range. She had a remote past medical history of left salpingo-oophorectomy. A CT scan of the abdomen revealed a pelvic mass measuring 15 x 9 x 9 cm with abdominal ascites (Figure 1). Patient underwent robotic-assisted laparoscopic hysterectomy and right salpingo-oophorectomy, omentectomy, pelvic lymph node, and peritoneal nodule biopsy.

**Figure 2:**
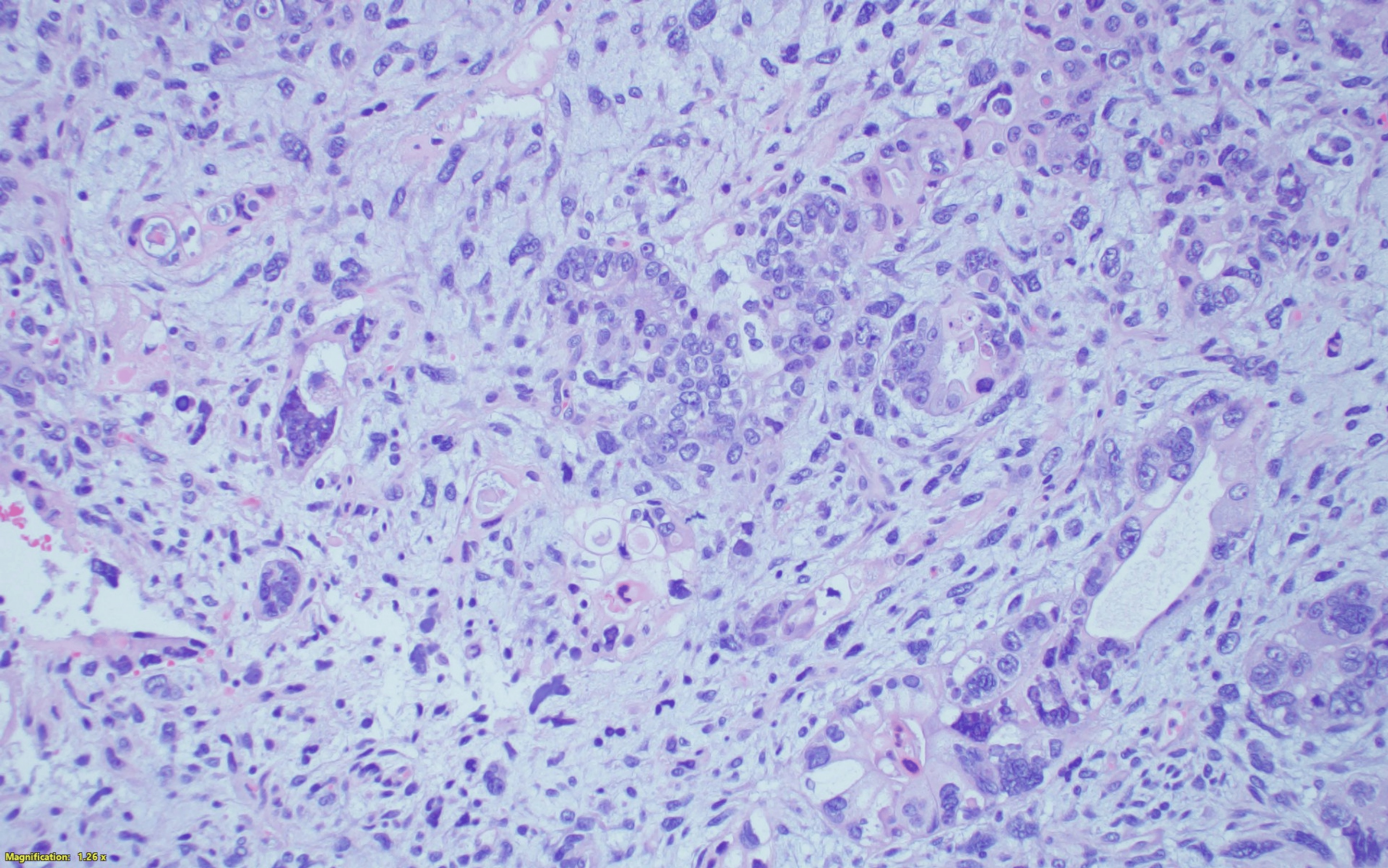
Hematoxylin and eosin (H&E) stain (400x) with carcinosarcoma showing malignant epithelial and stromal components.

Gross examination of the right ovarian mass revealed disrupted, friable, purple-grey, cystic soft tissue fragments with solid areas. Discrete nodules were not identified. The tumor was extensively sampled with 2 sections submitted per 1 cm. Microscopic evaluation showed a solid, cystic, biphasic neoplasm with malignant epithelial and stromal components and extensive necrosis. In solid areas, the epithelial component had a nonspecific, high-grade morphology, including solid, papillary, and acinar patterns. The stromal component showed increased cellularity, brisk mitotic activity, and moderate nuclear atypia (Figure 2). Heterologous elements were not identified.

**Figure 3:**
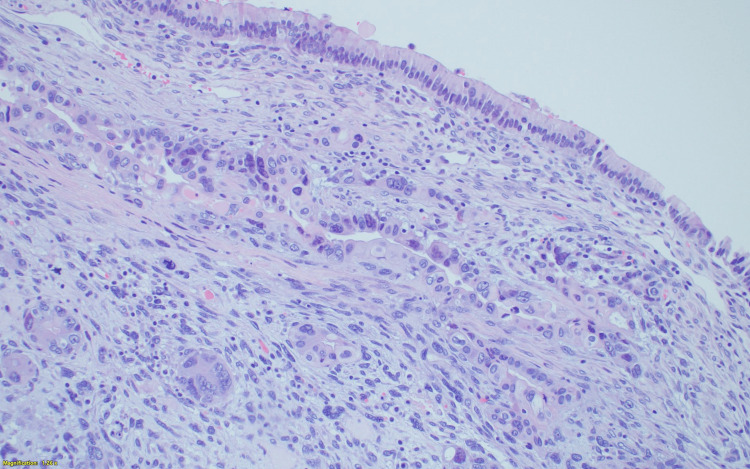
Hematoxylin and eosin (H&E) stain (400x) showing carcinosarcoma with associated mucinous cystadenoma component.

**Figure 4:**
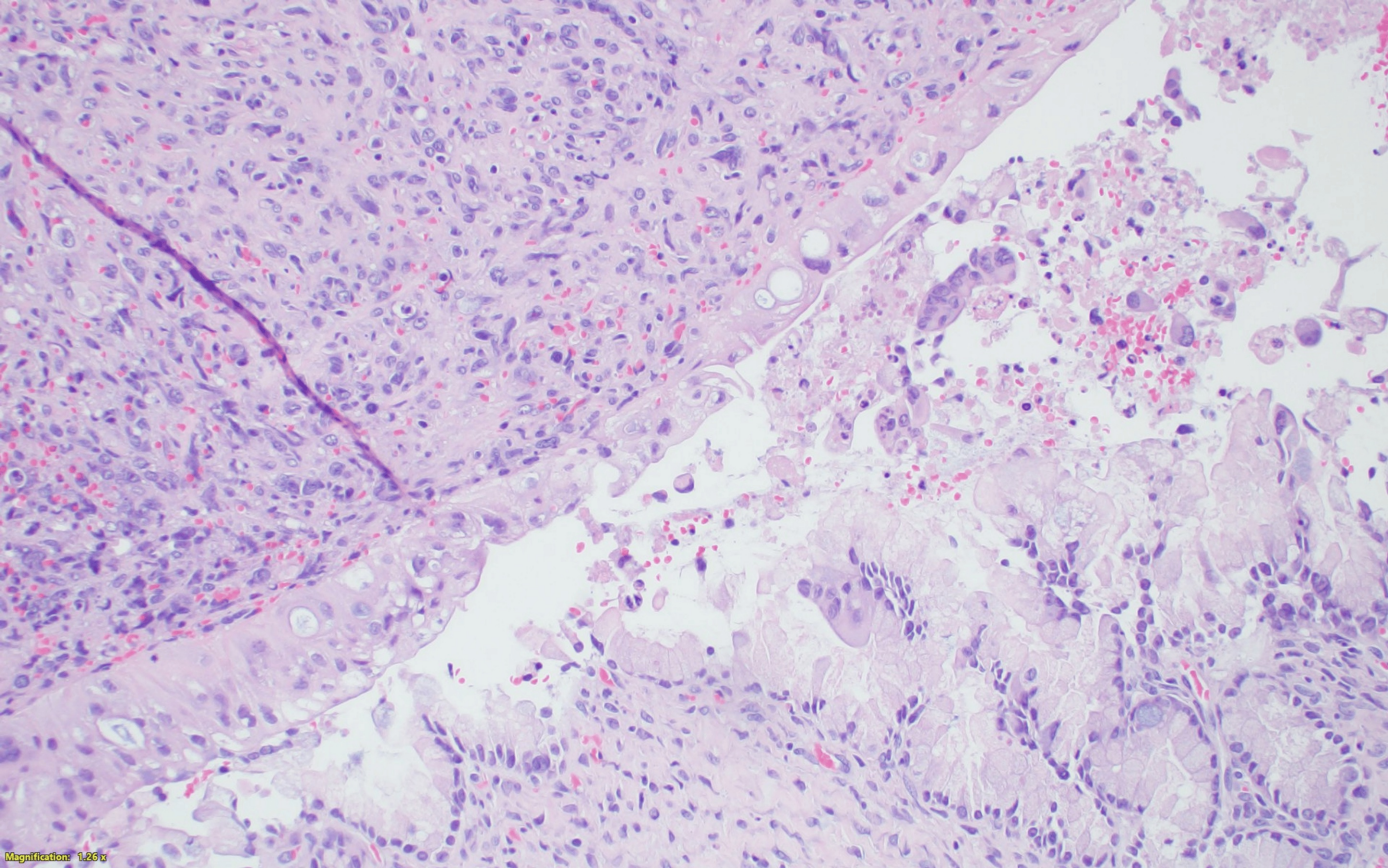
Hematoxylin and eosin (H&E) stain (400x) showing carcinosarcoma with associated mucinous borderline component.

The tumor was seen arising in a background of cystic areas showing a mucinous neoplasm (Figures 3-4) with components having morphology consistent with cystadenoma, borderline tumor, borderline tumor with intraepithelial carcinoma, and overt mucinous carcinoma with expansile and infiltrative components.

**Figure 5:**
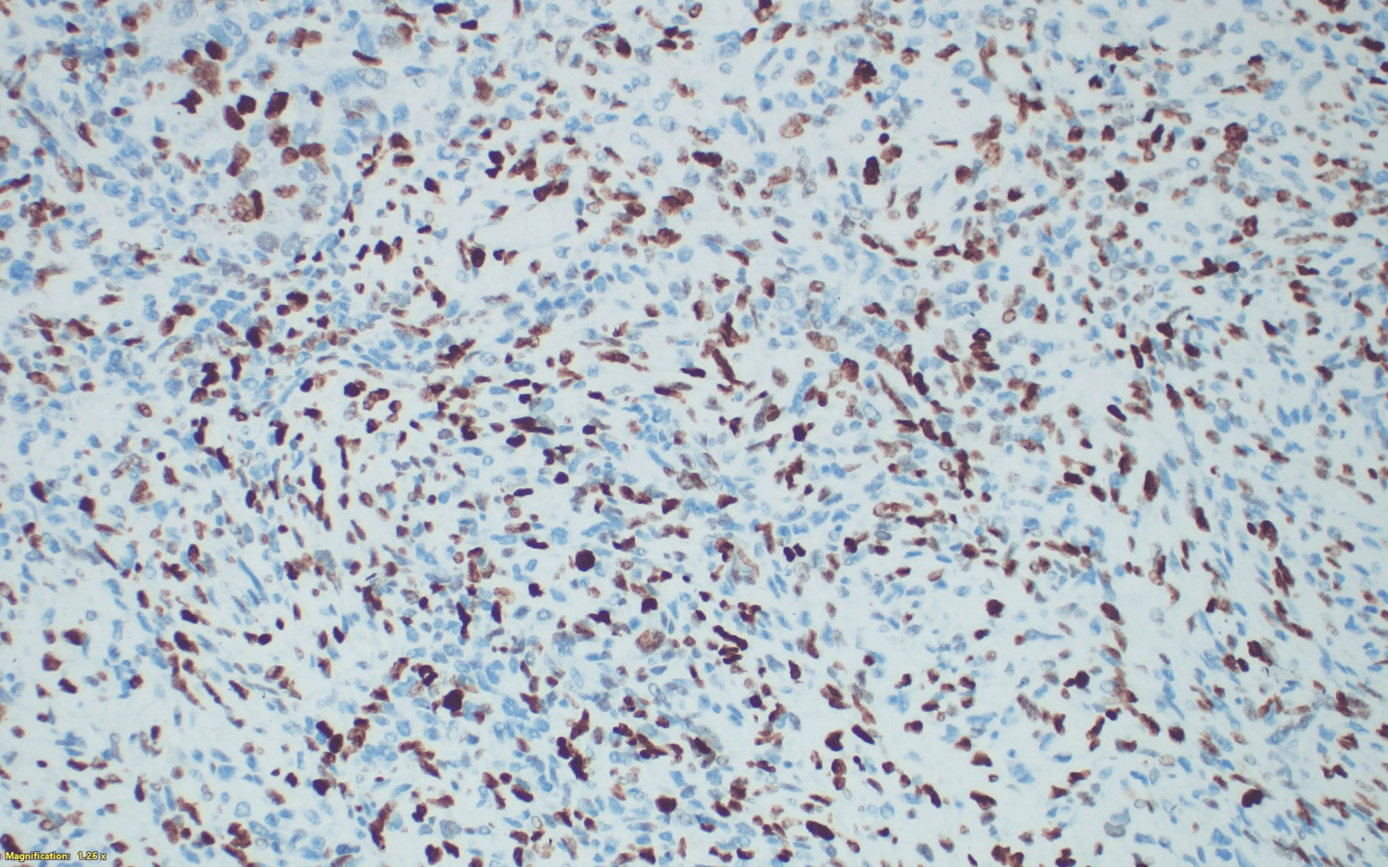
Ki-67 immunohistochemical stain (400x) showing increased expression in the stromal component.

**Figure 6:**
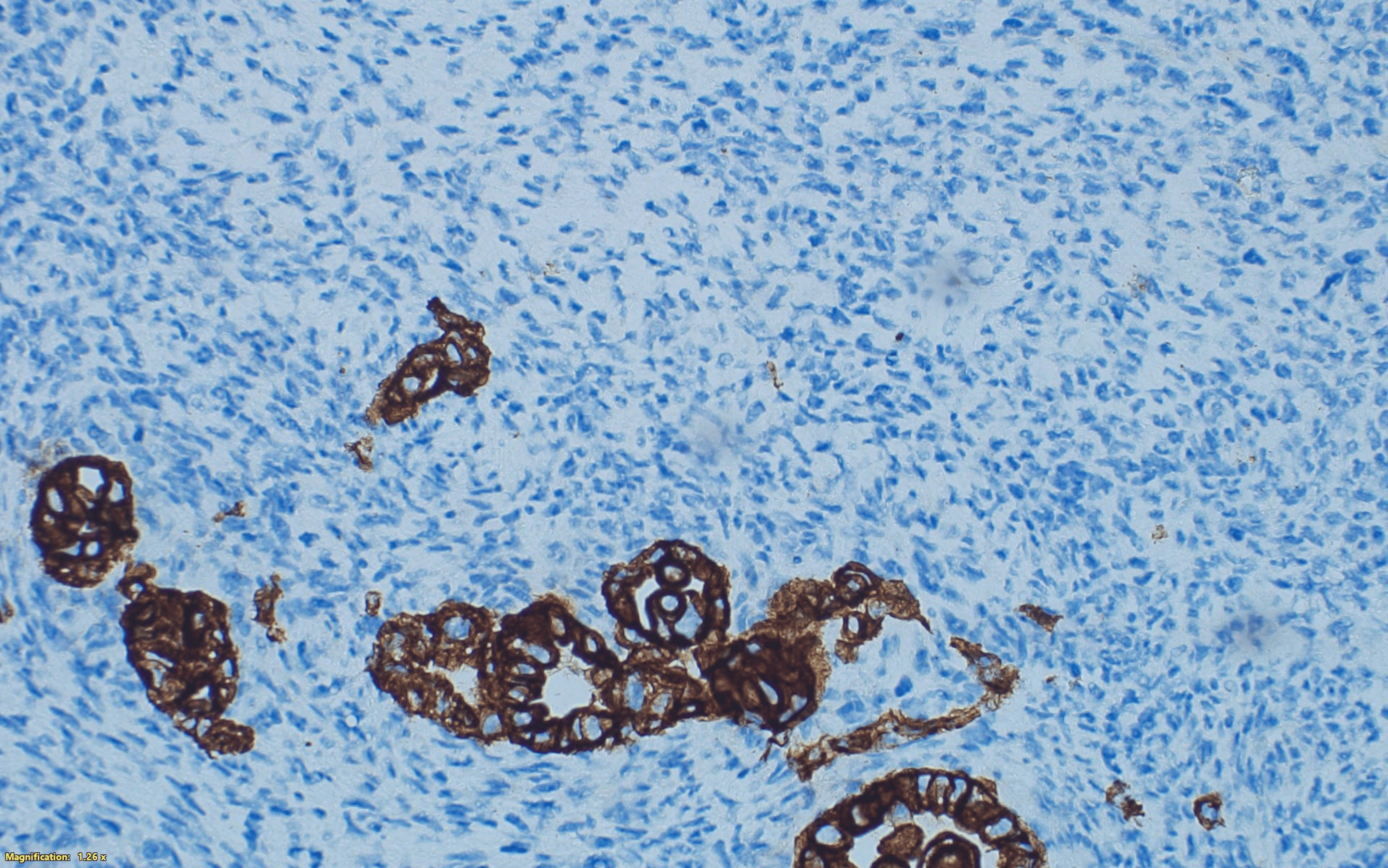
Pankeratin (CKAE1/AE3) immunohistochemical stain (400x) expression seen in epithelial component only.

Immunohistochemistry examination on the epithelial component of carcinosarcoma was notable for strong expression of CK7 and p53 and weak expression of CK20. Müllerian markers, including PAX8, WT1, ER, p16, and Naspin A, were negative. Ki-67 showed an increased proliferation index in the stromal component, i.e., more than 20% (Figure 5). Pankeratin expression was seen only in the epithelial component (Figure 6).

**Figure 7:**
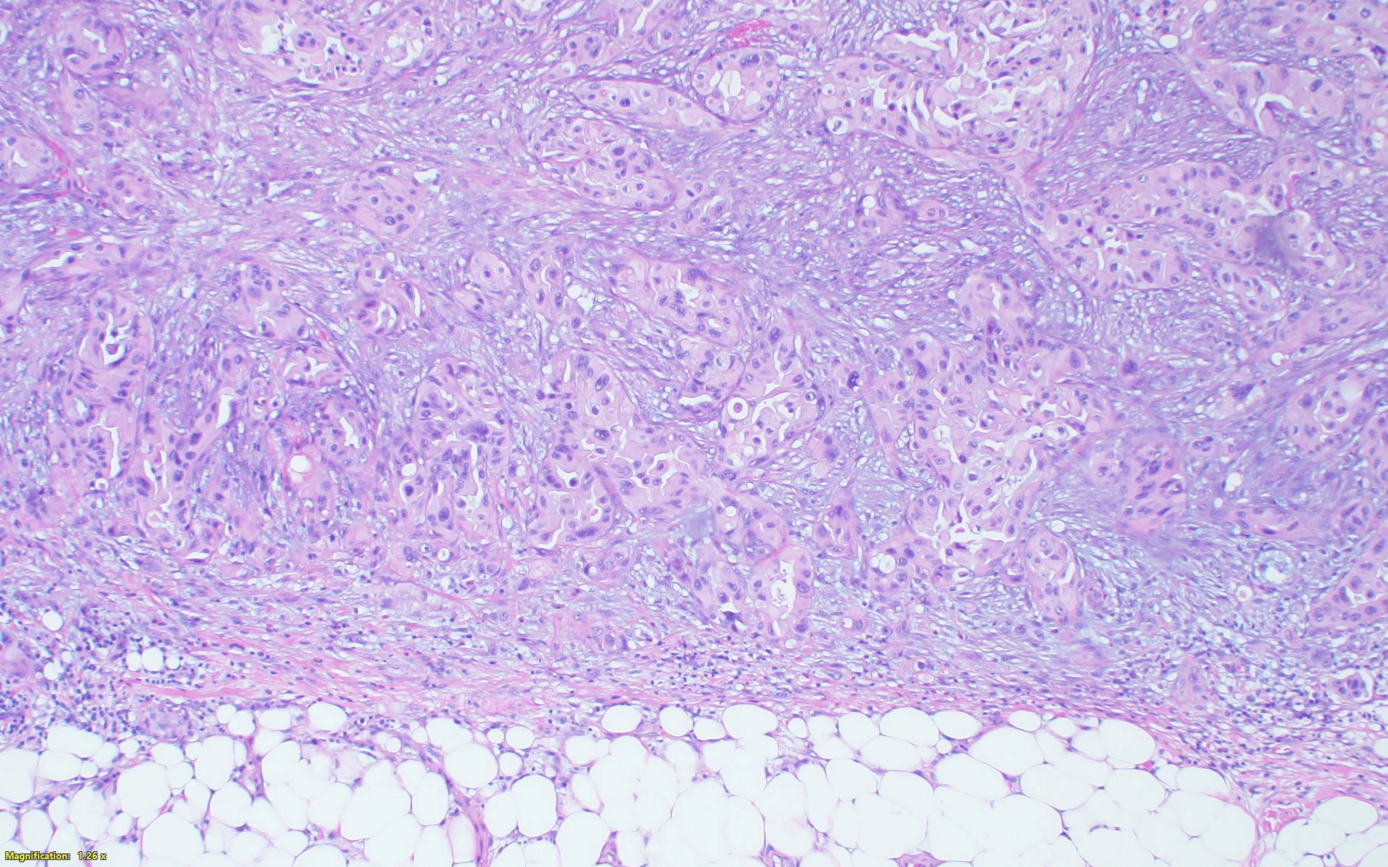
Hematoxylin and eosin (H&E) stain (400x) showing the omentum with metastatic carcinoma and desmoplastic stroma.

**Figure 8:**
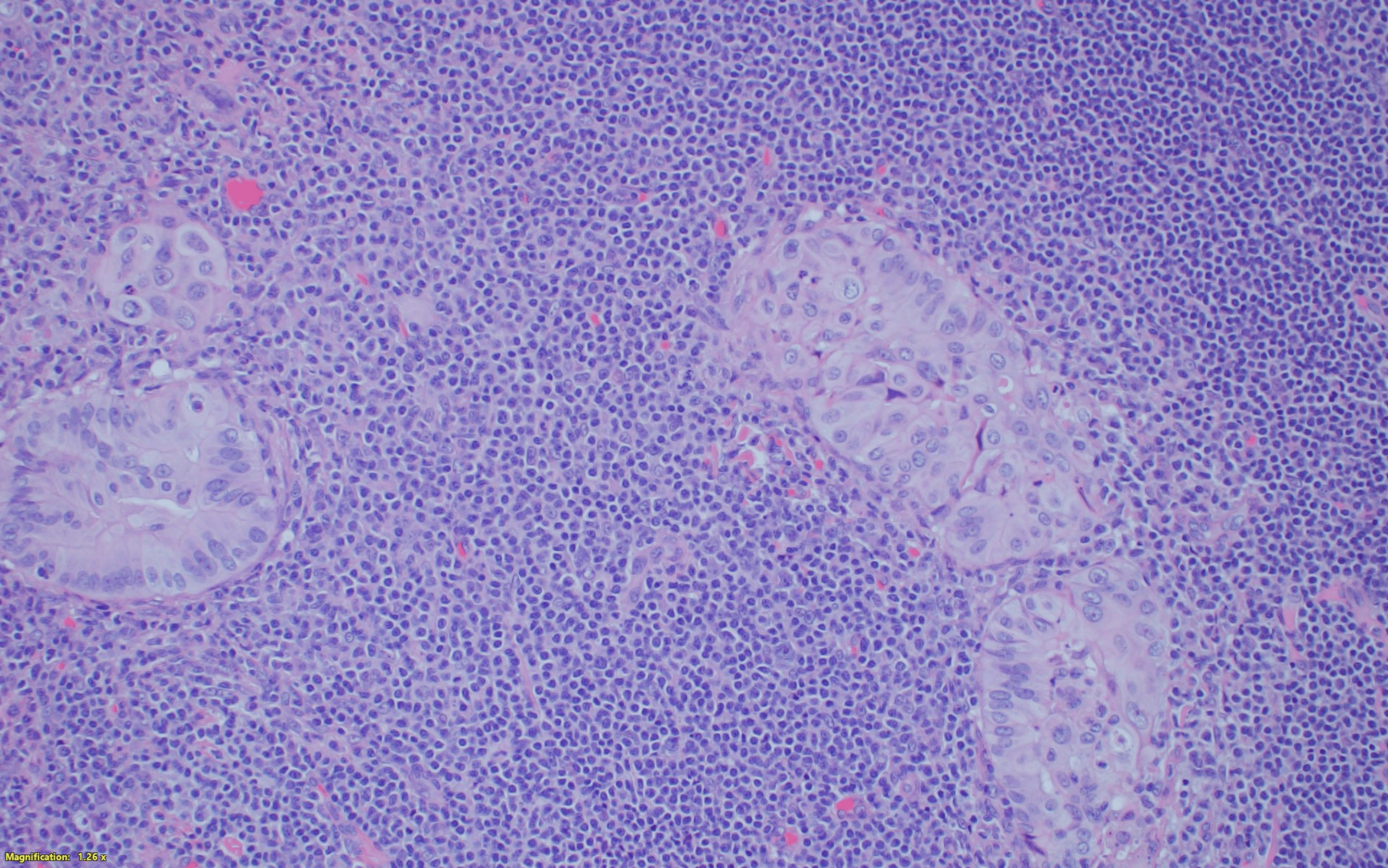
Hematoxylin and eosin (H&E) stain (400x) showing the lymph node with metastatic carcinoma.

The fallopian tube was benign; however, metastatic carcinoma without associated sarcoma was identified on the uterine serosa, peritoneum, omentum (Figure 7), and pelvic lymph node (Figure 8).

The tumor was positive for PDL1 (CPS: 1). Next-generation sequencing studies were performed and revealed the following pathogenic alterations: p53 (p.M246K), KRAS (p.G12D), and CDKN2A (p.H83Y). In addition, amplification was detected on the CRKL gene.

Based on our overall assessment, a diagnosis consistent with CSO originating in the background of a mucinous cystic neoplasm was rendered. Since the tumor was arising in the context of a mucinous neoplasm, diagnosis of a mural nodule was considered and excluded based on supporting arguments discussed in the following section. 

Clinically, the patient was staged as the International Federation of Gynecology and Obstetrics (FIGO) IIIA and treated with four cycles of carboplatin and paclitaxel followed by pembrolizumab. The patient remained stable at six months of diagnosis post surgery. However, she developed interval progression of disease at nine months with the development of ascites and pleural effusion, suggestive of pleural metastasis and increased size of peritoneal metastasis, which was confirmed by progression on a CT scan.

## Discussion

Mural nodules are rare, grossly circumscribed nodules associated with primary ovarian mucinous neoplasms [5]. Historically, they have been divided into three types: sarcoma-like, sarcomatous, and anaplastic nodules. Sarcoma-like nodules are typically small in size and display a heterogeneous morphology with varying degrees of pleomorphism, giant cells, inflammatory cells, and hemorrhage [6]. In contrast, sarcomatous and anaplastic nodules are larger, poorly circumscribed, and exhibit high-grade morphology. Anaplastic nodules have been reported to show a rhabdoid, sarcomatous, or pleomorphic pattern with some evidence of cytokeratin expression [7]. Sarcomatous nodules include various types of sarcoma, including fibrosarcoma, undifferentiated sarcoma, etc. [3]. Despite the distinctly described morphologies, mural nodules can have overlapping features, making subtypes hard to distinguish [5]. Rare case reports have also described carcinosarcomatous mural nodules, which are biphasic and contain both mucinous or non-mucinous carcinoma and differentiated or undifferentiated sarcoma [8-10]. In contrast to carcinosarcoma, however, these nodules were reported in younger patients and presented at an earlier stage [8].

In the present case, the tumor exhibited a distinct biphasic malignant morphology arising within a background of mucinous cystic neoplasm. Several findings support the diagnosis of carcinosarcoma over a mural nodule. First, the tumor had nonspecific, high-grade adenocarcinoma with an extensive sarcoma component that involved more than a discrete nodule. Second, pankeratin (AE1/AE3) immunoreactivity was confined to the epithelial component, effectively ruling out anaplastic differentiation. Third, the patient presented with advanced disease, and evaluation of extraovarian metastatic carcinoma showed a robust desmoplastic stromal reaction (Figure 7); however, the overtly malignant sarcomatous component was well represented only within the ovarian tumor. This pattern aligns with the pathology of CSO, in which metastases are predominantly epithelial.

Although overall features are consistent with CSO, this tumor had some noteworthy characteristics. First, the epithelial component showed a nonspecific morphology, although endometrial and serous subtypes are the most commonly presenting epithelial components in CSO, though other subtypes, including undifferentiated carcinomas, have been reported [11]. Second, no heterologous elements were identified in this tumor, despite being reported to occur in 50% of cases, most commonly rhabdoid and chondroid differentiation [12]. Third, next-generation sequencing revealed point mutations in p53, KRAS, and CDKN2A, which are non-specific. While p53 and KRAS mutations are known to occur in CSO, KRAS and CDKN2A mutations are frequently found in mural nodules associated with mucinous ovarian neoplasms [5]. Additionally, CRKL gene amplification was detected. Increased CRKL gene expression has been reported in epithelial ovarian cancers [13], though no exclusive role in the pathogenesis of CSO or mural nodules has been established.

Regarding prognosis, sarcoma-like mural nodules behave in a benign fashion with no effect on the prognosis of the associated mucinous tumor [6]. In contrast, overt sarcoma and anaplastic carcinoma in mural nodules carry a much worse prognosis, with mortality reported as early as three to eight months, although the study cohorts have been too small for robust statistical analysis [5]. Similarly, ovarian carcinosarcoma carries a poor prognosis, with median overall survival ranging from eight to 26 months depending on the study population and treatment received [11]. In the review of the largest confirmed cohort of CSO, median overall survival was 12.7 months [12]. In earlier stages, complete resection and platinum-containing adjuvant chemotherapy were associated with prolonged survival [14]. No relationship was found between histologic subtypes and prognosis, including heterologous differentiation [12]. CSO is also associated with worse outcomes compared to other ovarian cancers, including high-grade serous carcinoma [15]. Our patient's clinical trajectory illustrates this tumor's aggressive nature. Despite undergoing surgical resection and receiving adjuvant therapy with carboplatin/paclitaxel and pembrolizumab, she developed disease progression with malignant pleural effusion and expanding peritoneal metastases at nine months, falling within the survival intervals reported in the literature.

Although this paper adds to the existing literature on mural nodules and carcinosarcoma, it needs to be acknowledged that this is a single case report with a short patient follow-up; therefore, it has limited ability to define long-term patient prognosis. Also, given significant morphologic overlap between mural nodules and carcinosarcoma, establishing diagnostic criteria remains challenging.

## Conclusions

This case presents a rare occurrence of ovarian carcinosarcoma arising in the background of a mucinous cystic neoplasm. Key supporting features included a discrete biphasic malignant neoplasm extensively involving the ovarian mucinous tumor, pankeratin expression limited to the epithelial component only, and the presence of metastatic carcinoma without a sarcomatous component. It underscores the importance of extensive sampling and careful evaluation of complex ovarian neoplasms.
